# Aging impairs contraction-induced human skeletal muscle mTORC1 signaling and protein synthesis

**DOI:** 10.1186/2044-5040-1-11

**Published:** 2011-03-02

**Authors:** Christopher S Fry, Micah J Drummond, Erin L Glynn, Jared M Dickinson, David M Gundermann, Kyle L Timmerman, Dillon K Walker, Shaheen Dhanani, Elena Volpi, Blake B Rasmussen

**Affiliations:** 1 Division of Rehabilitation Sciences, University of Texas Medical Branch, Galveston, Texas, 77550 USA; 2 Department of Physical Therapy, University of Texas Medical Branch, Galveston, Texas, 77550 USA; 3 Department of Internal Medicine, University of Texas Medical Branch, Galveston, Texas, 77550 USA; 4 Sealy Center on Aging, University of Texas Medical Branch, Galveston, Texas, 77550 USA

## Abstract

**Background:**

Sarcopenia, the loss of skeletal muscle mass during aging, increases the risk for falls and dependency. Resistance exercise (RE) training is an effective treatment to improve muscle mass and strength in older adults, but aging is associated with a smaller amount of training-induced hypertrophy. This may be due in part to an inability to stimulate muscle-protein synthesis (MPS) after an acute bout of RE. We hypothesized that older adults would have impaired mammalian target of rapamycin complex (mTORC)1 signaling and MPS response compared with young adults after acute RE.

**Methods:**

We measured intracellular signaling and MPS in 16 older (mean 70 ± 2 years) and 16 younger (27 ± 2 years) subjects. Muscle biopsies were sampled at baseline and at 3, 6 and 24 hr after exercise. Phosphorylation of regulatory signaling proteins and MPS were determined on successive muscle biopsies by immunoblotting and stable isotopic tracer techniques, respectively.

**Results:**

Increased phosphorylation was seen only in the younger group (*P*< 0.05) for several key signaling proteins after exercise, including mammalian target of rapamycin (mTOR), ribosomal S6 kinase (S6K)1, eukaryotic initiation factor 4E-binding protein (4E-BP)1 and extracellular signal-regulated kinase (ERK)1/2, with no changes seen in the older group (*P >*0.05). After exercise, MPS increased from baseline only in the younger group (*P*< 0.05), with MPS being significantly greater than that in the older group (*P <*0.05).

**Conclusions:**

We conclude that aging impairs contraction-induced human skeletal muscle mTORC1 signaling and protein synthesis. These age-related differences may contribute to the blunted hypertrophic response seen after resistance-exercise training in older adults, and highlight the mTORC1 pathway as a key therapeutic target to prevent sarcopenia.

## Introduction

Maintenance of skeletal muscle mass is largely dependent on the dynamic relationship of muscle-protein balance, which is the relationship between protein synthesis and protein breakdown. A net negative protein balance is indicative of muscle atrophy, whereas a net positive balance yields an accrual of muscle proteins. In numerous disease states, such as HIV/AIDS, cancer, sepsis and renal failure, the rate of muscle-protein breakdown exceeds that of synthesis, and catabolism of muscle occurs, resulting in measurable atrophy [1-3]. Loss of muscle mass also occurs with the aging process (sarcopenia), although the atrophy of aging is not as severe as that seen in various disease states, as it arises over the span of several decades. Resting rates of muscle protein turnover have been investigated to explain the age-related loss of muscle mass, but most recent studies have failed to show any difference between young and older adults [4-8]. It is likely that the decrement in muscle mass with advanced age is due to inadequate stimulation of muscle-protein synthesis (MPS) after anabolic stimuli, such as resistance exercise or meal ingestion [4,9-14].

Resistance exercise is capable of increasing muscle mass through direct stimulation of MPS, which, over time, induces contractile protein accumulation and hypertrophy of individual muscle fibers. The fractional synthetic rate (FSR) of muscle proteins has been shown to be increased in as little as 1 hour [15] and for as long as 48 hours [16-18] after an unaccustomed, acute bout of resistance exercise. There is ample evidence supporting resistance-exercise training as a valuable intervention to induce muscle hypertrophy in young people. Less research has been conducted in aging populations, with some studies suggesting an age-related decline in the efficacy of resistance-exercise training (RET) to enhance muscle size and strength [19-21].

The reduced response to RET in older people may be due to an inability of the exercise bout to accelerate MPS [13,14]. An appealing candidate mechanism underlying the blunted anabolic effect of exercise in older persons is reduced activation of the mammalian target of rapamycin complex (mTORC)1. The mTORC1 signaling pathway is recognized as a key regulator of translation initiation and overall cell growth [22-25], and is important in the hypertrophic response after resistance exercise [26,27]. Our laboratory recently reported that mTORC1 activation is necessary for the resistance exercise-induced stimulation of MPS, as administration of rapamycin (a specific mTOR inhibitor) to humans before exercise prevented the contraction-induced increase in MPS [28]. After a bout of high-intensity resistance exercise, a rapid increase in the phosphorylation of extracellular signal-regulated kinase (ERK)1/2 and its downstream substrates was also shown [29-31]. Recent research has identified age-related differences in the mitogen-activated protein kinase (MAPK) signaling pathway, both at baseline and after exercise, which may also contribute to the differential response of skeletal MPS to resistance exercise in young and older adults [29,30].

An inability to fully activate the mTORC1 and other anabolic signaling pathways could be driving the blunted MPS response to an acute bout of resistance exercise in older adults, thereby hindering gains in muscle mass and strength with prolonged resistance training.

The aim of this study was to carry out a detailed and extended time-course investigation looking at the 24 hour response to an acute bout of resistance exercise in young and older adults to assess any age-related differences. We hypothesized that the older adults would have a blunted phosphorylation of several key intracellular proteins in the mTORC1 and the MAPK pathway signaling pathways, resulting in an impaired MPS response after resistance exercise.

## Results

### Study design

**Figure 1:**
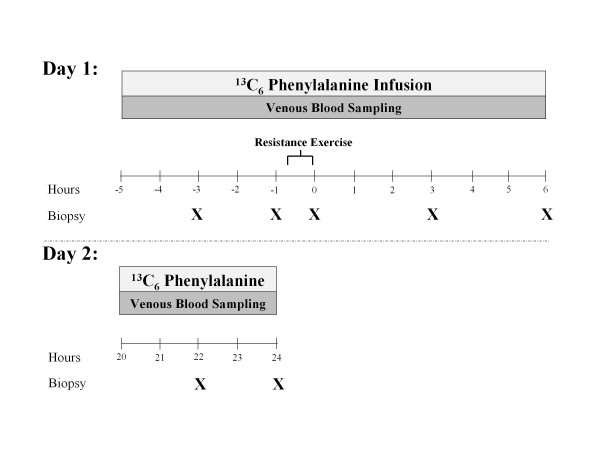
**Study design**. Blood was sampled throughout the study and muscle samples were taken at the times indicated (X). Exercise was performed after the second biopsy.

Blood was sampled throughout the study, and muscle samples were taken at the times indicated (X) in Figure 1. Exercise was performed after the second biopsy was taken.

### Plasma lactate, glucose and insulin

Plasma lactate values increased significantly during exercise in both groups (*P <*0.05; data not shown), with no differences between groups (*P >*0.05). Lactate concentration was similar in both groups throughout the duration of the study (*P >*0.05).

Plasma glucose levels decreased significantly at 6 hours after exercise in both groups (*P <*0.05; data not shown), with no differences between groups (*P >*0.05).

Plasma insulin levels were not different at baseline, and did not change significantly after exercise in either group (*P >*0.05; data not shown).

### mTORC1 signaling

**Figure 2:**
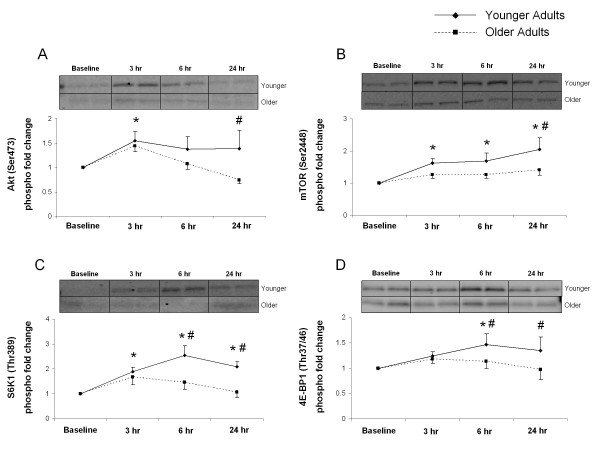
**Akt, mammalian target of rapamycin (mTOR), S6 kinase (S6K)1, eukaryotic initiation factor 4E-binding protein (4E-BP)1**. Data represent phosphorylation fold change of Akt at Ser473 (A), mTOR at Ser2448 (B), S6K1 at Thr389 (C) and 4E-BP1 at Thr37/46 (D) at baseline, 3, 6 and 24 hours after exercise. Representative immunoblot images are shown. Significantly different from *baseline (*P <*0.05); ^#^from older subjects (*P <*0.05).

Phosphorylation of Akt (Ser473) was increased at 3 hours after exercise in the younger group (*P <*0.05) (Figure 2A). The older group showed no significant changes in the phosphorylation of Akt (*P*> 0.05) (Figure 2A). However, at 24 hours after exercise, phosphorylation of Akt was significantly lower in the older group compared with the younger group (*P <*0.05) (Figure 2A).

Phosphorylation of mTOR (Ser2448) was increased at 3, 6 and 24 hours after exercise in the younger group (*P*< 0.05) (Figure 2B) with no significant changes observed in the older group after exercise (*P*> 0.05) (Figure 2B). mTOR phosphorylation in the older group was significantly lower than in the younger group (*P*< 0.05) at 24 hours after exercise (Figure 2B).

S6K1 (Thr389) phosphorylation increased significantly from baseline in the younger group at 3, 6 and 24 hours after exercise (*P*< 0.05) (Figure 2C). S6K1 phosphorylation did not change in the older group (*P*> 0.05) (Figure 2C), and at 6 and 24 hours after exercise, phosphorylation of S6K1 in the older group was lower than that of the younger group (*P*< 0.05) (Figure 2C).

Phosphorylation of eukaryotic initiation factor 4E-binding protein (4E-BP)1 (Thr37/46) was increased at 6 hours after exercise in the younger group compared with baseline values (*P*< 0.05) (Figure 2D). We observed no changes in phosphorylation in the older group (*P*> 0.05) (Figure 2D); however, at 6 and 24 hours after exercise, 4E-BP1 phosphorylation was significantly lower in the older than in the younger group (*P*< 0.05) (Figure 2D).

### MAPK signaling

**Figure 3:**
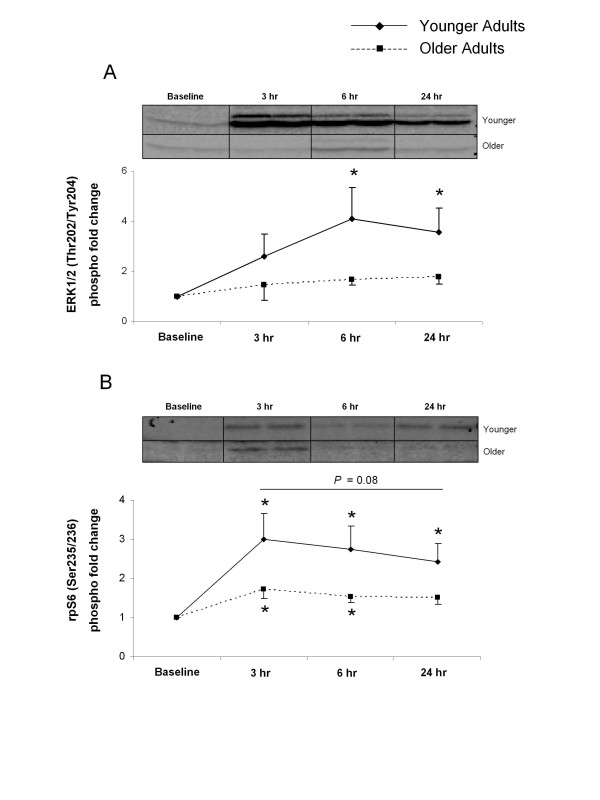
**Extracellular signal-regulated kinase (ERK)1/2 and ribosomal protein (rp)S6**. Data represent phosphorylation fold change of **(A) **ERK1/2 at Thr202/Tyr204 and **(B) **rpS6 at Ser235/236 at baseline, and 3, 6 and 24 hours after exercise. Representative immunoblot images are shown. *Significantly different from baseline (*P <*0.05).

The phosphorylation of ERK1/2 (Thr202/Tyr204) increased significantly from baseline at 6 and 24 hours after exercise in the younger group (*P*< 0.05) (Figure 3A). The older group showed no changes across time (*P*> 0.05) (Figure 3A). Phosphorylation of ribosomal protein (rp)S6 (Ser235/236) was increased at 3, 6 and 24 hours after exercise in the younger group (*P*< 0.05) (Figure 3B). rpS6 phosphorylation increased from baseline at 3 and 6 hours after exercise in the older group (*P*< 0.05) (Figure 3B). Although there were no significant differences between the groups, rpS6 phosphorylation was greater in the younger group, and this difference approached statistical significance (*P *= 0.08) (Figure 3B).

### Total protein content

**Figure 4:**
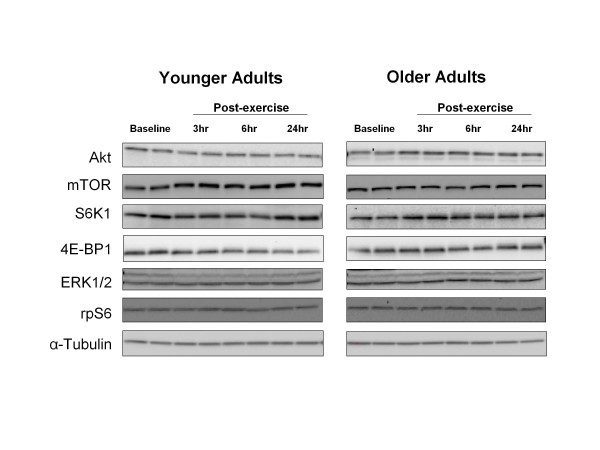
**Total protein content**. Representative immunoblot images for Akt, mammalian target of rapamycin (mTOR), S6 kinase (S6K)1,, eukaryotic initiation factor 4E-binding protein (4E-BP)1, extracellular signal-regulated kinase (ERK)1/2, ribosomal protein (rp)S6 and α-tubulin. Protein content did not change during postexercise recovery in either group (*P*> 0.05).

Total protein content of Akt, mTOR, S6K1, 4E-BP1, ERK1/2 and rpS6 did not change during the 24 hours of post-exercise recovery (*P >*0.05) (Figure 4).

### Additional regulators of mTORC1

In an attempt to identify why mTORC1 signaling was inhibited in older adults after resistance exercise, we measured several different proteins including insulin-like growth factor (IGF)-1, myostatin, Smad2 phosphorylation and adenine monophosphate protein kinase (AMPK)α phosphorylation. At each time point after exercise (*P >*0.05) for any of these four proteins, we could not find any differences that would have provided insight into the differential mTORC1 signaling response between young and older adults (data not shown). We also measured Pax7 mRNA expression (a marker of satellite cell activation) but found no differences between groups (*P*> 0.05; data not shown).

### MPS

**Figure 5:**
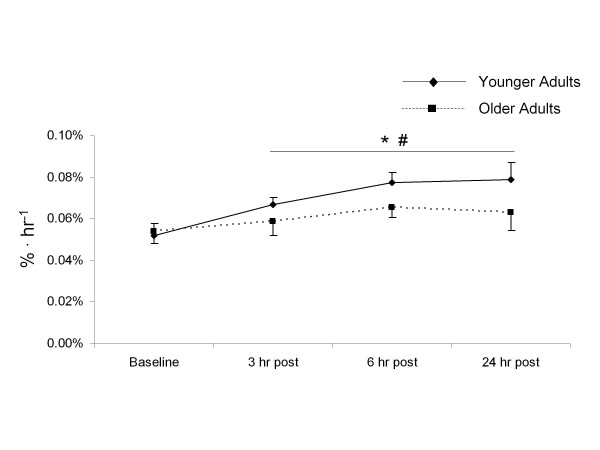
**Mixed muscle protein fractional synthetic rate (FSR)**. Muscle protein synthesis as expressed by the mixed muscle FSR (%/hour) in younger and older subjects at rest and at 3, 6 and 24 hours after exercise. *Main effect for time (*P <*0.05); #significantly different from older subjects (*P <*0.05).

The mixed muscle protein FSR was similar in both groups at rest (*P >*0.05) (Figure 5), but after exercise the rate of MPS significantly increased in both groups compared with baseline values (*P <*0.05) (Figure 5). However, MPS in the younger subjects increased to a greater extent than in the older subjects after exercise (*P <*0.05) (Figure 5).

### Association between mTORC1 signaling and MPS

**Figure 6:**
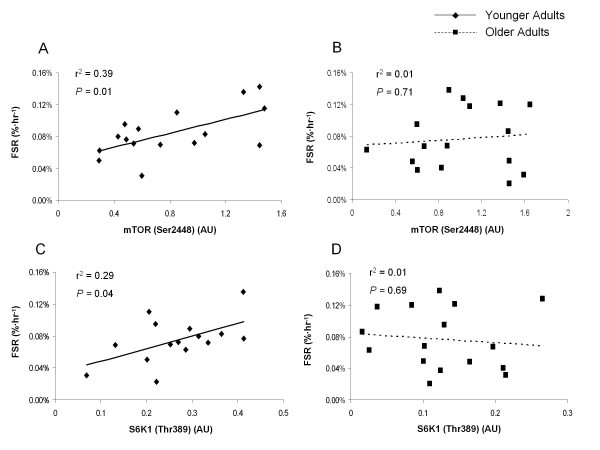
**Relationship between muscle protein fractional synthetic rate (FRS) and extent of phosphorylation of S6 kinase (S6K)1 and mammalian target of rapamycin (mTOR), at 24 hours after exercise**. There was a significant relationship (*P *= 0.01) between the degree of phosphorylation of mTOR (Ser2448) (AU) and mixed muscle protein fractional synthetic rate (%/hour) at 24 hours after exercise in **(A) **the young group only, with **(B) **no significant relationship seen in the older group (*P *= 0.71). There was a significant relationship (*P *= 0.04) between the degree of phosphorylation of S6K1 (Thr389) (AU) and mixed muscle protein fractional synthetic rate (%/hour) at 24 hours after exercise in **(C) **young group only, with **(D) **no significant relationship seen in the older group (*P *= 0.69). Note: some points overlaid.

The extent of phosphorylation of mTOR (Ser2448) was significantly related to the extent of MPS at 24 hours after exercise in young subjects only (*P *= 0.01) (Figure 6A), with no significant association seen in older subjects (*P *= 0.71) (Figure 6B).

The extent of phosphorylation of S6K1 (Thr389) was significantly related to the extent of MPS at 24 hours after exercise in young subjects only (*P *= 0.04) (Figure 6C), with no significant association seen in older subjects (*P *= 0.69) (Figure 6D).

## Discussion

In the current study, we assessed age-related differences in translation initiation signaling and mixed muscle protein FSR in the 24 hours period after an acute bout of resistance exercise. We report, for the first time, a detailed time-course study of the differential aging response after high-intensity resistance exercise. Recent studies have shown a blunted initial anabolic response to exercise with aging [13,14]; however, we studied molecular signaling and MPS data over an extended recovery period, detailing more fully the age-related differences after exercise. We found that MPS and associated translational signaling through the mTORC1 and MAPK pathways are upregulated at multiple post-exercise time points in younger subjects, with a depressed response in both intracellular signaling and MPS after an acute bout of resistance exercise in older subjects.

High-intensity resistance exercise is well established as a potent stimulus for MPS and hypertrophy in young adults [13,14,16,19,32-34], and we have shown that a single bout of resistance exercise at 70% 1 repetition maximum (1RM) increases MPS during exercise recovery [15]. In the current study, we observed an increase in the rate of MPS at all post-exercise time points in our younger subjects. The rate of protein synthesis was highest in younger subjects at 24 hours after exercise, with a 53% increase from baseline measures. We observed a much less robust change in the rate of MPS in older subjects after exercise, which is in agreement with previous research showing a blunted protein synthesis response in the very acute (< 4 hours) post-exercise period for older people [13,34]. Recent research has suggested a reduced hypertrophic response to resistance training in older adults [19-21,35], perhaps because an acute bout of resistance exercise cannot adequately stimulate MPS in this group, leading to a blunted accrual of muscle proteins over time after repeated bouts of exercise.

We also assessed the expression of several key signaling proteins in this study. Several proteins in the mTORC1 signaling pathway, including Akt, mTOR, S6K1 and 4E-BP1, showed increased phosphorylation after exercise in younger subjects. Increased phosphorylation of these proteins is indicative of improved translation initiation. Similarly, we previously found an increase in phosphorylation of several mTORC1-associated proteins after a bout of high-intensity resistance exercise [15], and we recently reported that the contraction-induced increase in MPS is dependent on mTORC1 activation in human muscle through the use of a specific mTOR inhibitor [28]. Several studies have shown that the gradual activation of mTORC1 and its downstream target S6K1 in the recovery phase after high-intensity resistance exercise [15,36,37] is associated with increased protein synthesis [25,26,38,39]. The degree of S6K1 phosphorylation in the first few hours after an acute bout of high-intensity resistance exercise has been strongly correlated with the percentage change in muscle mass after several weeks of high-intensity RET in both rodents [26] and humans [27]. The lack of phosphorylation of mTORC1-associated proteins after exercise in older subjects may partly explain the blunted MPS response.

Similar to our findings of an age-related decline in mTORC1 signaling, Kumar *et al. *recently reported an age-related differential response to resistance exercise, with older subjects failing to show improved phosphorylation of two key targets of mTOR, S6K1 and 4E-BP1, at 1 hour after exercise [13]. However, our findings contrast with another recent study: Mayhew *et al. *did not find significant age-related decrements in translational signaling, although they did observe a blunted protein-synthesis response [14]. These differences may be due to different exercise protocols, and some age-related differences may have been missed because sampling of muscle was performed only at 24 hours after exercise in the Mayhew study [14]. The current study expands upon the findings from these studies, showing for the first time that older adults continue to display a blunted signaling response in the 24 hour period after resistance exercise, with only younger adults showing significant increases in phosphorylation, most of which peak at 6 hours after exercise.

We also found a positive correlation between the extent of phosphorylation of both mTOR and S6K1 and MPS in human skeletal muscle at 24 hours after exercise in young adults. These findings support previous research in rats and humans, showing that the extent of S6K1 phosphorylation predicted total muscle accretion after resistance training [26,27]. Our findings support an integral role for S6K1 in stimulating MPS after resistance exercise, as short-term changes in both predict muscle protein accrual over repeated bouts of exercise. We also noted a lack of correlation between mTOR and S6K1 phosphorylation and MPS in older subjects, which corresponds with the blunted MPS response to exercise that we found. The lack of association between signaling and MPS may help explain why recent studies have noted a blunted hypertrophy response after RET in older men [19-21,35].

Although mTORC1 signaling has been shown to be vital in regulating protein synthesis after exercise, other pathways are also involved (for example, the mitogen-activated protein kinase (MAPK) pathway). ERK1/2 can activate the eukaryotic initiation factor 4E, a translation initiation factor, through its downstream target MAPK-interacting kinase 1 [29,30,40]. In addition, ERK1/2 is also capable of phosphorylating rpS6 (via p90 ribosomal S6K 1) on its Ser235/236 regulatory site [41,42]. However, rpS6 can also be phosphorylated by S6K1 on both its Ser235/236 and Ser240/244 regulatory sites [43]. rpS6 is associated with increased translation of mRNAs involved in the synthesis of ribosomal proteins, along with elongation and initiation factors necessary for translation [44,45]. After a bout of high-intensity resistance exercise, phosphorylation of ERK1/2 and its downstream substrates rapidly increases [29-31]. Recent research also illustrates the age-related differences at baseline and after exercise in the MAPK-associated proteins, a potential mechanism that may help explain the age-related discrepancy in skeletal MPS response after resistance exercise [29,30]. In agreement with those studies, we previously found that the phosphorylation of ERK1/2 is blunted in older adults after a bout of high-intensity resistance exercise and essential amino acid ingestion [31]. In the current study, we observed an increase in ERK1/2 phosphorylation at 6 and 24 hours after exercise in young but not older subjects. The phosphorylation of rpS6 was also significantly increased in younger subjects at all post-exercise time points, and phosphorylation of rpS6 tended to be greater in younger than in older subjects (*P *= 0.08). These data indicate that activation of both the mTORC1 and MAPK signaling pathways probably contributes to the MPS response after resistance exercise, and in this study we found a blunted response in both signaling pathways after resistance exercise in older adults.

Although our data provide evidence for dysregulation in mTORC1 signaling after resistance exercise in older adults, we are unable to definitively determine the factor(s) responsible for the reduced mTORC1 signaling response in older adults in the present study. In an effort to address this question, we examined several upstream regulators of mTORC1, including IGF-1, myostatin, Smad2 and AMPK phosphorylation, but we did not detect any group differences for any of these proteins after exercise. We did find that Smad2 phosphorylation increased at 6 and 24 hours after exercise in both groups (*P*< 0.05), but because there were no group differences, it does not seem likely that the myostatin/TGF-β-Smad2/3 signaling pathway and AMPK are responsible for the reduced mTORC1 signaling response in older adults after resistance exercise.

## Conclusions

In summary, an acute bout of high-intensity resistance exercise stimulates MPS and enhances phosphorylation of proteins in both the mTORC1 and MAPK signaling pathways during the 24 hour post-exercise recovery period in young but not older adults. Consequently, concurrent activation of both the mTORC1 and MAPK signaling pathways seems to be an important cellular mechanism for enhanced MPS after resistance exercise, and the inability to activate these pathways probably contributes to the impaired MPS response associated with aging. The reduced gains in muscle mass and strength after resistance training in older adults may be caused by the impaired response to an acute bout of exercise. With aging, skeletal muscle mTOR signaling seems to be fully functional, as other mTORC1 regulators such as insulin [46] and amino acids [31] are capable of activating mTORC1 signaling and protein synthesis. Therefore, mTORC1 is an important pathway to target in future evidence-based rehabilitation interventions to counteract sarcopenia.

## Methods

The study was approved by the Institutional Review Board of the University of Texas Medical Branch and carried out in accordance with principles of the Declaration of Helsinki. All subjects gave informed written consent before participating in the study.

### Subjects

We studied 16 young (eight men, eight women; mean ± SD age 27 ± 2 years) and 16 older (eight men, eight women; 70 ± 2 years) subjects. Demographic information is given in Table 1. All subjects were healthy and physically active but were not currently engaged in an exercise training program. Screening of subjects included clinical history, physical examination, stress test, laboratory investigations (complete and differential blood counts, liver and kidney function tests, coagulation profile, fasting blood glucose and oral glucose tolerance test, thyroid-stimulating hormone, lipid profile, urinalysis, drug screening and tests for hepatitis B and C viruses and HIV ), and electrocardiography. On two separate occasions (> 7 days apart) and > 7 days before the study was conducted, each subject was tested for muscle strength by measuring their 1RM on a leg extension machine (Cybex-VR2, Medway, MA, USA) located within the exercise laboratory of the Institute for Translational Sciences Clinical Research Center (ITS-CRC) of the University of Texas Medical Branch. The higher of the two 1RM values obtained was used to determine the weight (70% of 1RM) for the resistance exercise portion of the study.

**Table 1:** Subject characteristics

	Younger	Older
Subjects, *n *(M:F)	16 (8:8)	16 (8:8)
Characteristics^a^		
Age, years	27 ± 2	70 ± 2^d^
Weight, kg	70.2 ± 3.1	66.9 ± 3.0
Height, cm	167.2 ± 3.0	165.9 ± 2.5
BMI ^b^, kg/m^2^	25.1 ± 0.9	24.2 ± 0.6
Body fat, %	28.3 ± 2.2	31.5 ± 2.0
Lean mass, kg	48.4 ± 3.2	43.7 ± 2.7
Bilateral leg extension 1RM ^c^, kg	92.9 ± 9.0	62.4 ± 5.6^d^
1RM/lean mass	1.9 ± 0.1	1.4 ± 0.1^d^

^a^Values are means ± SE.

^b^BMI; = body mass index,

^c^1RM; = 1 repetition maximum.

^d^Significantly different from younger group (*P <*0.05).

### Study design

Each subject was admitted to the ITS-CRC the day before the exercise study, and dual-energy X-ray absorptiometry (Hologic QDR 4500 W; Bedford, MA, USA) was performed to measure body composition and lean mass. The subjects were then fed a standard dinner (12 kcal/kg of body weight; 60% carbohydrate, 20% fat and 20% protein) and a snack at 22.00 hours, prepared by the Bionutrition Division of the ITS-CRC. The subjects were studied after an overnight fast under basal conditions, and they refrained from exercise for 48 hours before study participation. All subjects were studied during the same time of day (04.00-16.00 for infusion study 1. and 04.00-09.00 for infusion study 2).

### Infusion study 1

The morning of the infusion study, at 04.00, an 18 G polyethylene catheter was inserted into an antecubital vein for tracer infusion. Another 18 G polyethylene catheter was inserted retrogradely in a hand vein of the contralateral arm, which was kept in a heated pad for arterial blood sampling. After a background blood sample was drawn, a primed continuous infusion of L-[*ring*-^13^C_6_] phenylalanine (Sigma-Aldrich, St. Louis, MO, USA) was begun, and maintained at a constant rate until the end of the experiment (Figure 1). The priming dose for the labeled phenylalanine was 2 μmol/kg and the infusion rate was 0.05 μmol/kg/min. At 2.5 hours after the initiation of the tracer infusion, the first muscle biopsy was taken from the lateral portion of the vastus lateralis of the leg, with the biopsy site at between 150 and 250 mm from the mid patella. The biopsy was taken using a 5 mm Bergström biopsy needle under sterile procedure and local anesthesia (1% lidocaine). Muscle tissue was immediately blotted and frozen in liquid nitrogen, and stored at -80°C until analysis. Two hours after the first biopsy, a second biopsy was taken from the same incision. The biopsy needle was inclined at a different angle so that the second biopsy was taken approximately 50 mm apart from the first. This method has been previously used by us [15,31,47] and others [48-50]. After the second muscle biopsy, subjects were seated on the leg-extension machine to begin the exercise portion of the study. Subjects completed a warm-up set of 10 repetitions at 45% 1RM and eight sets of 10 repetitions at 70% 1RM with 3 minutes of rest between each set. Immediately after the last set, a third muscle biopsy was taken from the same incision. Total time for the exercise period was approximately 45 minutes. Blood was obtained at selected intervals over the next 3 hours, and muscle biopsies were sampled from a new incision, approximately 50 mm proximal to the first, at 3 and 6 hours after exercise. After collection of the fifth muscle biopsy, infusion study 1 was concluded and subjects were given a standard lunch. Subjects were also fed a dinner and snack similar to that on the previous night, before an overnight fast in preparation for the second infusion protocol.

### Infusion study 2

The morning of the second infusion study, at 04.00, an 18 G polyethylene catheter was inserted into an antecubital vein for tracer infusion. Another 18 G polyethylene catheter was inserted retrogradely in a hand vein of the contralateral arm, which was kept in a heated pad for arterial blood sampling. After a background blood sample was drawn, a primed continuous infusion of L-[*ring*-^13^C_6_] phenylalanine (Sigma-Aldrich) was begun, and maintained at a constant rate until the end of the experiment (Figure 1). The priming dose for the labeled phenylalanine was 2 μmol/kg and the infusion rate was 0.05 μmol/kg/min. At 2.5 hours after the initiation of the tracer infusion, the first muscle biopsy was taken from the lateral portion of the vastus lateralis of the contralateral leg from infusion study 1 with the biopsy site between 150 and 250 mm from the mid patella. The biopsy was taken using a 5 mm Bergström biopsy needle under sterile procedure and local anesthesia (1% lidocaine). Muscle tissue was immediately blotted and frozen in liquid nitrogen, and stored at -80°C until analysis. Two hours after the first biopsy, a second biopsy was taken from the same incision. The biopsy needle was inclined at a different angle so that the second biopsy was taken approximately 50 mm apart from the first. After collection of the second muscle biopsy, infusion study 2 was concluded.

### SDS-PAGE and western blot analysis

Details of the immunoblotting procedures have been published previously [15]. Briefly, approximately 30 to 50 mg of frozen tissue was homogenized (1/9 w/v), separated by centrifugation at 3400 g for 10 minutes at 4°C, followed by the removal of the supernatant. Total protein concentrations were determined by using the Bradford assay (Smartspec Plus spectrophotometer; Bio-Rad, Hercules, CA, USA). The supernatant was diluted (1:1) in a sample buffer mixture containing 125 mmol/L Tris (pH 6.8), 25% glycerol, 2.5% SDS, 2.5% B-mercaptoethanol and 0.002% bromphenol blue, and then boiled for 3 minutes at 100°C. Each sample (50 μg of total protein) was loaded in duplicate on a 7.5% or 15% polyacrylamide gel (Criterion; Bio-Rad), and separated by electrophoresis (150 V for 1 hour). A molecular weight ladder (Precision Plus protein standard; Bio-Rad) and a normalization control were also included on each gel. After electrophoresis, proteins were transferred to a polyvinylidene difluoride membrane (Bio-Rad) at 50 V for 1 hour. Blots were incubated with a single primary antibody overnight at 4°C (see below). The next morning, blots were incubated in secondary antibody for 1 hour at room temperature. Chemiluminescent solution (ECL plus; Amersham BioSciences, Piscataway, NJ, USA) was applied to each blot. After 5 minutes of incubation, optical density measurements were obtained with a phosphoimager (Bio-Rad) and densitometric analysis was performed (Quantity One software, version 4.5.2; Bio-Rad). Membranes containing phosphodetected proteins were stripped of primary and secondary antibodies, then re-probed for total protein. Total protein was determined for each blot, which did not change from baseline over the course of the experiment (Figure 4). An internal loading control (α-tubulin) was also assessed to ensure that a traditional housekeeping gene product was not changing over time (Figure 4). However, for consistency with our previous publications [15,51-53], data are presented as phosphorylation status relative to the standard loading control that was loaded on every gel, and then expressed as a fold change from baseline.

### Antibodies

The following primary antibodies were used: phospho-Akt (protein kinase B) (Ser473), phospho-mTOR (Ser2448), phospho-p70 S6K1 (Thr389), phospho-rpS6 (Ser235/236), phopsho-4E-BP1 (Thr37/46), phospho-ERK1/2 (Thr202/Tyr204), phospho-Smad2 (Ser465/467), phospho-AMPK (Thr172), total Akt, total mTOR, total p70 S6K1, total rpS6, total 4E-BP1, total ERK1/2, total Smad2, total AMPK and total α-tubulin (all from Cell Signaling, Beverly, MA, USA), total myostatin (Millipore, Billerica, MA, USA) and total IGF-1 (Santa Cruz Biotechnologies, Santa Cruz, CA, USA). All antibodies were used in a dilution of 1:1000 except for phospho-S6K1 (1:500) and α-tubulin (1:30,000). Anti-rabbit IgG horseradish peroxidase-conjugated secondary antibody (1:2000) was purchased from Amersham Biosciences (Piscataway, NJ, USA). Fold change mRNA expression for Pax7 was measured using the 2^-ΔΔCt ^method as reported previously [53] using β2-Microglubulin as the housekeeping gene.

### Plasma glucose/lactate/insulin

Plasma glucose and lactate concentrations were measured using an automated lactate analyzer (YSI, Yellow Springs, OH, USA). Plasma concentrations of insulin were determined (Millipore) via ELISA at selected time points according to the manufacturer's instructions.

### Muscle fractional synthesis rate

Muscle intracellular free amino acids and muscle proteins were extracted as previously described [54,55]. Muscle intracellular free concentration and enrichment of phenylalanine was determined by gas chromatography-mass spectrometry (GC-MS, 6890 Plus GC, 5973N MSD, 7683 autosampler, Agilent Technologies, Palo Alto, CA, USA) using appropriate internal standard (L-[^13^C_9_, ^15^N]phenylalanine) [54,55]. Mixed muscle protein-bound phenylalanine enrichment was analyzed by GC-MS after protein hydrolysis and amino acid extraction [54,55], using external standard curve [56]. We calculated the fractional synthetic rate of mixed muscle proteins (FSR) by measuring the incorporation rate of the phenylalanine tracer into the proteins (ΔEp/*t*) and using the precursor-product model to calculate the synthesis rate:

$$\text{FSR}=(\Delta \text{Ep/}t)/\left[\left({\text{E}}_{\text{M1}}+{\text{E}}_{\text{M2}}\right)\text{/2}\right]\times \text{6}0\times \text{1}00,$$

where ΔEp is the increment in protein-bound phenylalanine enrichment between two sequential biopsies, *t *is the time between the two sequential biopsies, and E_M1 _and E_M2 _are the phenylalanine enrichments in the free intracellular pool in the two sequential biopsies. Data are expressed as percentage per hour.

### Statistical analysis

All values are expressed as means ± SE. Comparisons were performed using ANOVA with repeated measures, the effects being group (younger, older) and time (baseline, and 3, 6 and 24 hours after exercise). *Post hoc *testing was performed using Bonferroni correction where appropriate. If a test of normality and/or equal variance failed, simple transformations were performed. Where appropriate, correlations were tested by assessing the existence of a linear fit between the extent of phosphorylation of mTORC1-associated proteins and MPS. Significance was set at *P *≤ 0.05. All analyses were performed with SigmaStat software (version 11.0; Systat Software Inc, San Jose, CA, USA).

## Competing interests

The authors declare that they have no competing interests.

## Authors' contributions

CSF participated in the design of the study, collected the data, analyzed the data, performed the statistical analysis, and wrote and edited the manuscript. MJD participated in the design of the study, collected the data and edited the manuscript. ELG, JMD, DMG, KLT and DKW collected the data and edited the manuscript. SD collected the data. EV participated in study design and coordination and edited the manuscript. BBR conceived of the study, participated in its design and coordination and helped to draft the manuscript. All authors read and approved the final manuscript.
